# Supplementary material for: Longitudinal resting-state fMRI of awake mice during habituation: stress, head motion, and functional connectivity
Source: Front Neurosci. 2026 Feb 18;20:1773151. doi: 10.3389/fnins.2026.1773151 (PMC12956629; doi:10.3389/fnins.2026.1773151)
Supplement: Supplementary file 1 [file Data_Sheet_1.docx]

Supplementary Material

# Supplementary Figures


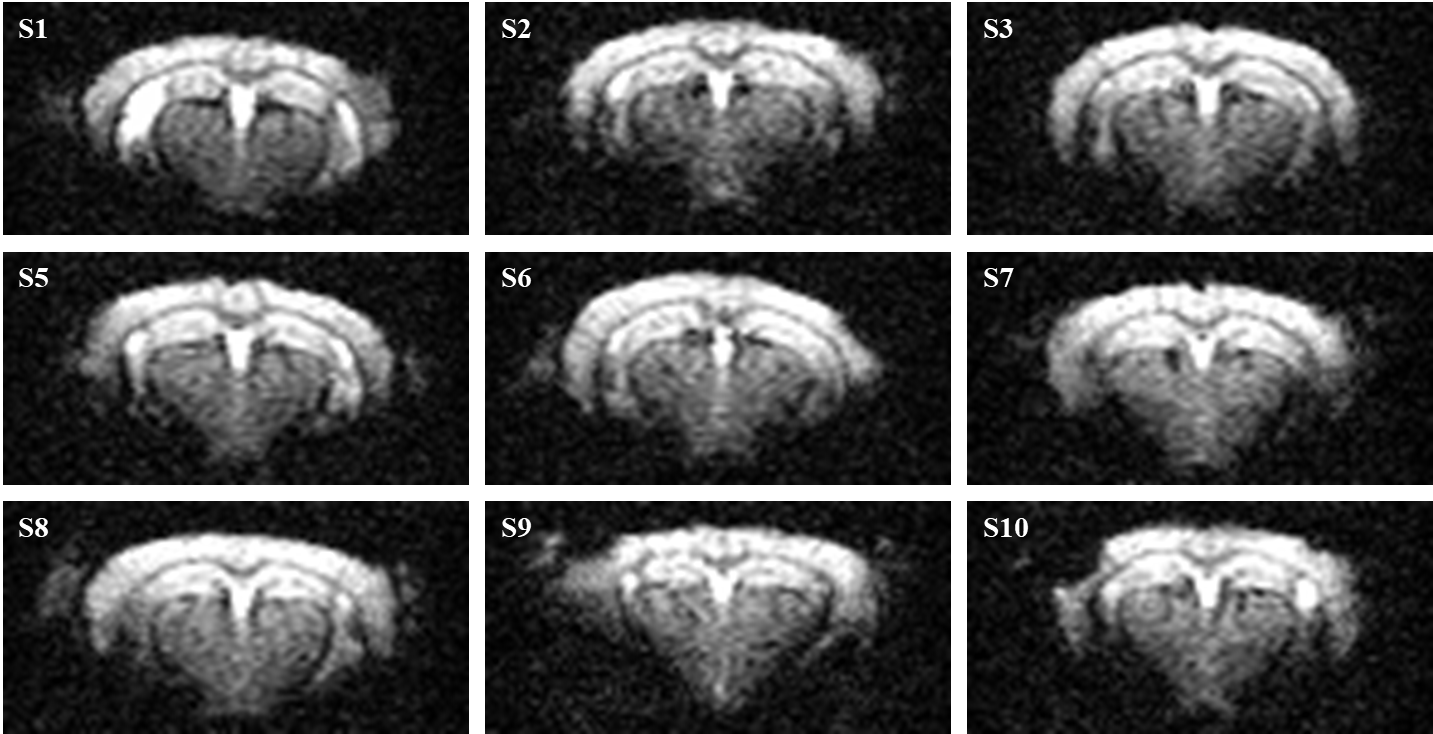


**Supplementary Figure 1. Representative raw EPI images from all 9 animals.** Each image shows a representative slice (slice #9) from the first volume of the EPI dataset acquired during the first trial of the first session on Day 1. The number in the upper left corner of each figure indicates the subject ID.


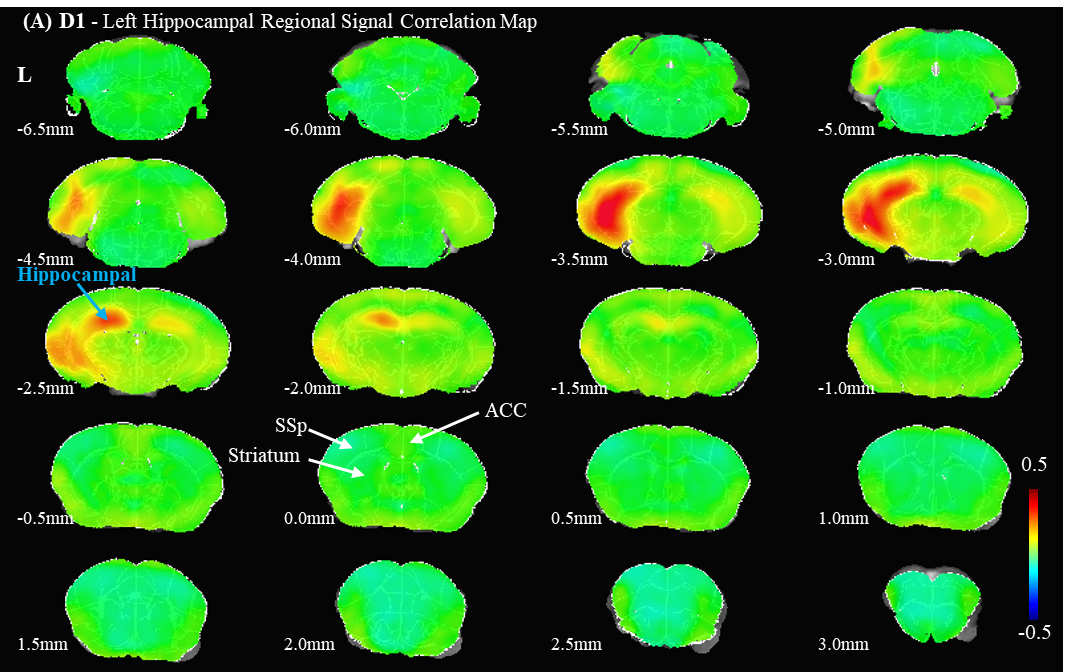


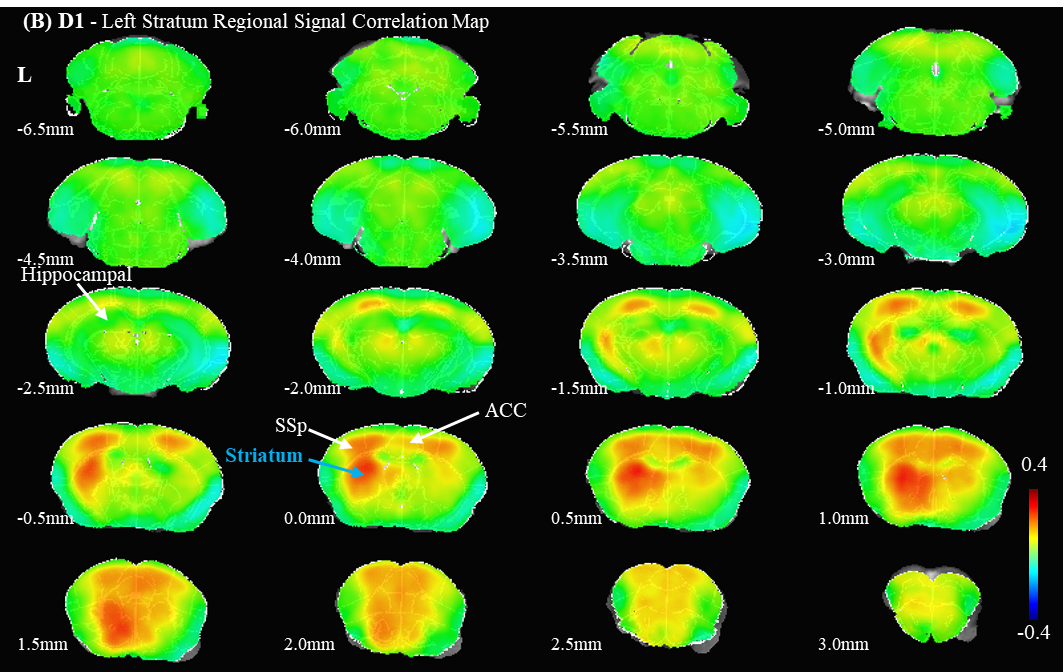


**Supplementary Figure 2. Hippocampal region- and Striatum-seed based cross-correlation maps of the resting-state fMRI on the initial habituation day (n = 9).** (A) Cross-correlation maps based on the left hippocampal seed. (B) Cross-correlation maps based on the left striatal seed. ACC, anterior cingulate; SSp, primary somatosensory.
